# miR‐1254 inhibits progression of glioma in vivo and in vitro by targeting CSF‐1

**DOI:** 10.1111/jcmm.14981

**Published:** 2020-01-28

**Authors:** Xin Li, Shiqi Kong, Yingxiao Cao

**Affiliations:** ^1^ Department of Neurosurgery The First People's Hospital of Shenyang Shenyang Liaoning China; ^2^ Department of Neurosurgery Xingtai People's Hospital Xingtai Hebei China

**Keywords:** CSF‐1, glioma, invasion, migration, miR‐1254

## Abstract

The role of miRNAs (microRNAs) has been implicated in glioma initiation and progression, although the inherent biochemical mechanisms still remain to be unravelled. This study strived to evaluate the association between CSF‐1 and miR‐1254 and their effect on advancement of glioma cells. The levels of miR‐1254 in glioma cells and tissues were determined by real‐time RT‐PCR. Proliferation, apoptosis and cell cycle arrest, invasion and migration, were assessed by CCK‐8 assay, colony formation assay, flow cytometry, transwell assay and wound‐healing assay, respectively. The targeted relationship between miR‐1254 and CSF‐1 was confirmed by dual‐luciferase reporter assay. The effects of CSF‐1 on cellular functions were also assessed. The in vivo effect of miR‐1254 on the formation of a tumour was explored by using the mouse xenograft model. We found in both glioma tissues and glioma cells, the down‐regulated expressions of miR‐1254 while that of CSF‐1 was abnormally higher than normal level. The target relationship between CSF‐1 and miR‐1254 was validated by dual‐luciferase reporter assay. The CSF‐1 down‐regulation or miR‐1254 overexpression impeded the invasion, proliferation and migratory ability of U251 and U87 glioma cells, concurrently occluded the cell cycle and induced cell apoptosis. Moreover, in vivo tumour development was repressed due to miR‐1254 overexpression. Thus, CSF‐1 is targeted directly by miR‐1254, and the miR‐1254/CSF‐1 axis may be a potential diagnostic target for malignant glioma.

## 1 INTRODUCTION

In adults, gliomas is the most rapidly progressive and lethal brain tumour, which among all brain malignancies exhibits the highest rate of mortality.^1^, ^2^ Although multimodality treatments, such as surgical resection ensuing radiotherapy and chemotherapy, are available, the prognosis is still poor for glioma patients, with only 14.6 months as the median survival time.^3^ The recurrence after treatment is caused because of highly aggressive, quick proliferation of gliomas which facilitates their infiltration into adjacent normal brain tissue.^4^, ^5^ However, the mechanisms leading to the advancement of malignancy and glioma cells are still uncovered to a large extent, and a better understanding is imperative to discovering more robust therapeutic strategies.

MicroRNAs (miRNA) are non‐coding, endogenous small RNA molecules that aid in gene regulation.^6^, ^7^ They bind to partly complementary sequences present on the 3'‐UTRs (3'‐untranslated regions) of their cognate target genes and induce mRNA degradation or inhibit translation.^8^ In the human genome, more than 2500 miRNAs have been identified, which potentially regulate > 50% of all human protein‐coding gene expressions.^9^, ^10^ A common trait of human malignancies, including glioma, is the aberrant expression of miRNAs which have been shown to regulate key pathological processes, such as cell angiogenesis, EMT (epithelial‐mesenchymal transition), metastasis, proliferation and apoptosis.^11^, ^12^, ^13^ Several miRNAs find their role in the initiation and advancement of glioma and could potentially act as markers for the diagnosis and therapy of this malignancy.^14^ For example, a down‐regulation of miR‐365 is observed in glioma which targets phosphoinositide‐3‐kinase (PI3K) regulatory subunit 3 and leads to inhibition of glioma cell migration, invasion and proliferation,^15^ whereas upregulation of miR‐93 is observed in glioma, which activates the PI3K/AKT signalling pathway and promotes glioma cell proliferation.^16^ MiR‐1254 may have a crucial part in multiple malignancy‐related processes as it was found down‐regulated in colorectal cancer and cervical cancer, although till date, its associated molecular mechanisms remain unclear.^17^, ^18^ Therefore, we aimed to detect miR‐1254 expression, as well as examine its biological roles and underlying mechanisms, in glioma.

CSF‐1 (colony‐stimulating factor), also called the M‐CSF (macrophage colony‐stimulating factor), can facilitate cell proliferation, growth and differentiation of monocyte‐macrophages, as well as maintenance of its biological functions.^19^, ^20^ Recently, few studies have shown the vital role of CSF‐1 in tumorigenesis, which has been established in leukaemia, lung cancer, lymphoma, ovary and breast.^21^, ^22^ Besides, increased metastatic potential and a poor prognosis in breast cancer cells was indicated through the nuclear staining of CSF‐1.^23^ In canine mammary cancer cells, CSF‐1R has been recognized as a promoter of migration, proliferation and invasion.^24^ Stalling CSF‐1R and CSF‐1 would limit metastasis and primary tumour growth by depleting TAMs (tumour‐associated macrophages).^25^ The growth and metastasis of tumours is reduced by targeting TAMs via the inhibition of CSF‐1R, the key macrophage signalling pathway, and this outcome is now further being validated by clinical trials.^22^, ^26^ The CSF‐1 and its receptor co‐expression in samples of metastatic ovarian cancer is a predictor of poor outcome.^27^ Hence, CSF‐1/CSF‐1R signalling is deemed a possible target for progression and metastasis of cancer. The relation of miR‐1254 and CSF‐1 are not well understood in previously study. Thus, we investigated the effect of miR‐1254/CSF‐1 axis on glioma.

In this study, we evaluated the glioma cell processes in terms of the function of miR‐1254. We determined the miR‐1254 levels in glioma samples and assessed the relationship between miR‐1254 and CSF‐1 to evaluate its association with progression of glioma cells.

## 2 MATERIALS AND METHODS

### 2.1 Specimens of human tissues

Samples of human glioma tissues and that of healthy brain were procured after the operation from The First People's Hospital of Shenyang. Pathologists classified the histological grade of each sample as per WHO criteria. No patients with surgical resection had undergone radiotherapy or chemotherapy prior to the operation. After obtaining informed consent from patients, tumour tissues were collected and quickly frozen in liquid nitrogen until further use. The Ethics Committee of The First People's Hospital of Shenyang gave consent to the use of human specimens for experiments.

### 2.2 Cell culture

The Cell Bank of Chinese Academy of Sciences (Shanghai, China) supplied with the cell lines for human glioma A172, H4, LN229, U87, U251 and U118. NHAs (normal human astrocytes) were procured from Lonza (Switzerland) and cultured as per supplied instructions. Each cell line was maintained in DMEM (Dulbecco's modified Eagle's medium) along with sodium pyruvate and foetal bovine serum (FBS; 10%) supplementation, high glucose and antibiotics (100 ng of streptomycin/mL and 100 U of penicillin/mL). The cells were kept at 37℃ in a humid atmosphere with 5% CO_2_.

### 2.3 Quantitative real‐time PCR

From the specimens of glioma cell lines and human glioma tissues, total RNA was extracted using TRIzol reagent from Invitrogen as per supplied instructions.^28^, ^29^ The levels of miR‐1254 were evaluated through the stem‐loop RT primer assay, and small nuclear RNA U6 was used to normalize the assay. Amplification of cDNAs was performed by real‐time PCR on a 7900HT system using SYBR Premix DimerEraser from Takara, as per instructions. The 2^−ΔΔCt^ analysis method was used for measuring relative gene expression. The primers used in this study are as follows: hsa‐miR‐1254, Forward: 5′‐AGCCTGGAAGCTGGAGCCTGCAGT‐3′, Reverse: 5′‐GCGAGCACAGAATTAATACGAC‐3′; U6, Forward: 5′‐CTCGCTTCGGCAGCACA‐3′, U6, Reverse: 5′‐AACGCTTCACGAATTTGCGT‐3′; CSF‐1, Forward: 5'‐ACCCCTCCACCCTCTCTG‐3', Reverse: 5'‐CTGCCCCTTCACTTGCTG‐3'; β‐actin, Forward: 5′‐CTCACCATGGATGATGATATCGC‐3′, Reverse: 5'‐AGGAAT‐CCTTCTGACCCATGC‐3′.

### 2.4 Cell transfection

In six‐well plates, seeding of U87 and U251 cells was performed and grown at 37℃ in 5% CO_2_ for one full day until 40%‐50% confluence. Mimics of miR‐1254 (miR‐1254 group), miR‐1254 inhibitor (anti‐miR‐1254 group) and the mock group (negative control oligonucleotides) were procured from Sangon Biotech (China). To aid in cell transfection, lipofectamine 2000 from Invitrogen was used. The eukaryotic expression vector containing complete sequence of CSF‐1 cDNA was labelled as pcDNA3.1‐CSF‐1 and was prepared by Sangon Biotech (China). The vector pcDNA3.1‐CSF‐1 and pcDNA3.1‐shCSF‐1 (TG305200, Origene, China) were transfected along with Lipofectamine 2000 into glioma cells as per supplied protocol and transferred to complete medium 6 hours after transfection. After 24 hours, the level of miR‐1254 and CDF‐1 was analysed by real‐time PCR and Western blotting, respectively.

### 2.5 Cell‐counting assay

In 96‐well plates transfected cells (5 × 10^3^ cells/well) were incubated for 1, 2 or 3 days. Then, a CCK‐8 (cell counting kit‐8; Dojindo Laboratories) was used to detect the rate of cell proliferation as per provided instructions.

### 2.6 Flow cytometric analysis

Fixing of the collected cells was performed for 1 hour in ethanol (75%) at 4℃ and washed thrice with PBS (phosphate‐buffered saline) prior to adding 1 mL PBS with propidium iodide (PI; 40 μg) and RNase A. The cell cycle distribution was analysed by flow cytometry using FACSCalibur from Becton Dickinson (USA) and cell apoptosis using FITC Annexin V Apoptosis Detection Kits from Becton Dickinson. The analyses of data were performed using FACS Diva (Becton Dickinson). Each experiment was performed three times, independently.

### 2.7 Colony formation assay

This was performed as previously described.^30^, ^31^ Per well six‐well plate, the indicated cells were seeded and maintained for 14 days. Then, 4% paraformaldehyde was used to fix visible colonies for 0.5 hour and stained with crystal violet (0.1%) for 120 minutes. The cell activity was estimated as the colony‐forming efficiency after transfection.

### 2.8 Wound‐healing assay

After one full day of transfection, cells were cultured in six‐well plates after seeding till a confluence of 90% was reached. Then using a 200‐μL sterile pipette tip, the cell layer was scratched. The medium, suspension cells and cell debris were removed, and serum‐free medium was added and kept in incubator for one full day and then cell migration was visualized and photographed.

### 2.9 Transwell assay

For overnight, the Matrigel (BD, USA) was melted at 4℃, blended with thrice the volume of serum‐free medium and 50 μL of this solution was added per well into 24‐well transwell chambers. After incubation for 0.5‐hour, DMEM (containing 10% FBS) was added to the lower chambers, and collected cells (4 × 10^4^) in total were seeded in the upper chambers of the incubator and kept for another one full day. The membrane containing the cells on the upper surface were wiped lightly using swabs of cotton; the cells that moved across the polycarbonate membrane were methanol‐fixed and stained with crystal violet (0.1%). The invaded cells were enumerated from five random fields under a microscope. The experiments were performed thrice, independently.

### 2.10 Dual‐luciferase reporter assay

One of the targets of miR‐1254 is CSF‐1, as mentioned in TargetScan (http://www.targetscan.org/). Mutation in CSF‐1‐3'‐UTR was performed through multisite directed mutagenesis. These 3'‐UTRs (mutated and wild‐types) were cloned in pmirGLO vector from Promega (WI, USA). The cells were cotransfected with mimics of miR‐1254 and 3'‐UTRs (CSF‐1‐wt or CSF‐1‐mut). After one full day, the relative luciferase activity was detected.

### 2.11 Western blotting

The extraction of protein and Western blotting were performed as described previously.^32^ In short, lysis of specimens was carried out on ice for 0.5 hour in the assay buffer for radio immunoprecipitation from KenGEN (China). Centrifugation of lysates was performed at 14 000 × *g* and 4℃ for 15 minutes. After collecting the supernatant, concentration of protein was estimated using the bicinchoninic acid assay kit from Pierce (Rockford, USA). After separating equal amounts of protein on 10% SDS‐PAGE by electrophoresis, their transfer was performed onto PVDF (polyvinylidene difluoride membranes) from Millipore Corporation (USA) and blocked for 2 hours. Then, they were incubated overnight with a primary antibody, and then with respective secondary antibodies, and visualized using ECL (enhanced chemiluminescence reagents). The primary antibodies are as follows: Cyclin D1 (55 506, Cell Signaling Technology), CDK4 (12 790, Cell Signaling Technology), β‐acitn (A5441, Sigma) and CSF‐1 (TA806568, Thermo Fisher, USA).

### 2.12 Xenograft

The Department of Laboratory Animal Science, China Medical University (Shenyang, China) provided with BALB/c nude mice (n = 12; all male) at 6‐week average age and weight 16‐20 g and were arbitrarily and equally categorized into 2 groups as control group (mice injected with U87‐Mock cells) and U87 cells transfected with AgomiR‐1254 (Guangzhou, China). Experiments on animals adhered stringently to the directives of the Animal Ethics Committee of The First People's Hospital of Shenyang. Mice from the 2 groups were injected subcutaneously with 100 μL (1 × 10^6^ cells) of U87‐miR‐1254 or U87‐Mock cell sap in the right axillary. One full week after injection, the subcutaneous tumours were measured using a Vernier caliper every 2 days. The volume size was determined as follows: Volume = 1/2(L × W^2^). Each mouse was killed at the end of four weeks post‐injection and tumours were extracted for evaluation.

### 2.13 Immunohistochemistry

Tumour samples of mouse xenograft were immunohistochemically stained with antibodies against CSF‐1 (Abcam) and Ki67 (Abcam) as mentioned previously.^33^

### 2.14 Statistical analysis

All experiments were carried out thrice, independently. Data were assessed for pairwise comparison by the Student's *t* test, or ANOVA for multivariate analysis. Differences at *P* < .05 were deemed significant statistically.

## 3 RESULTS

### 3.1 Repression of miR‐1254 in glioma cells and tissues

**Figure 1:**
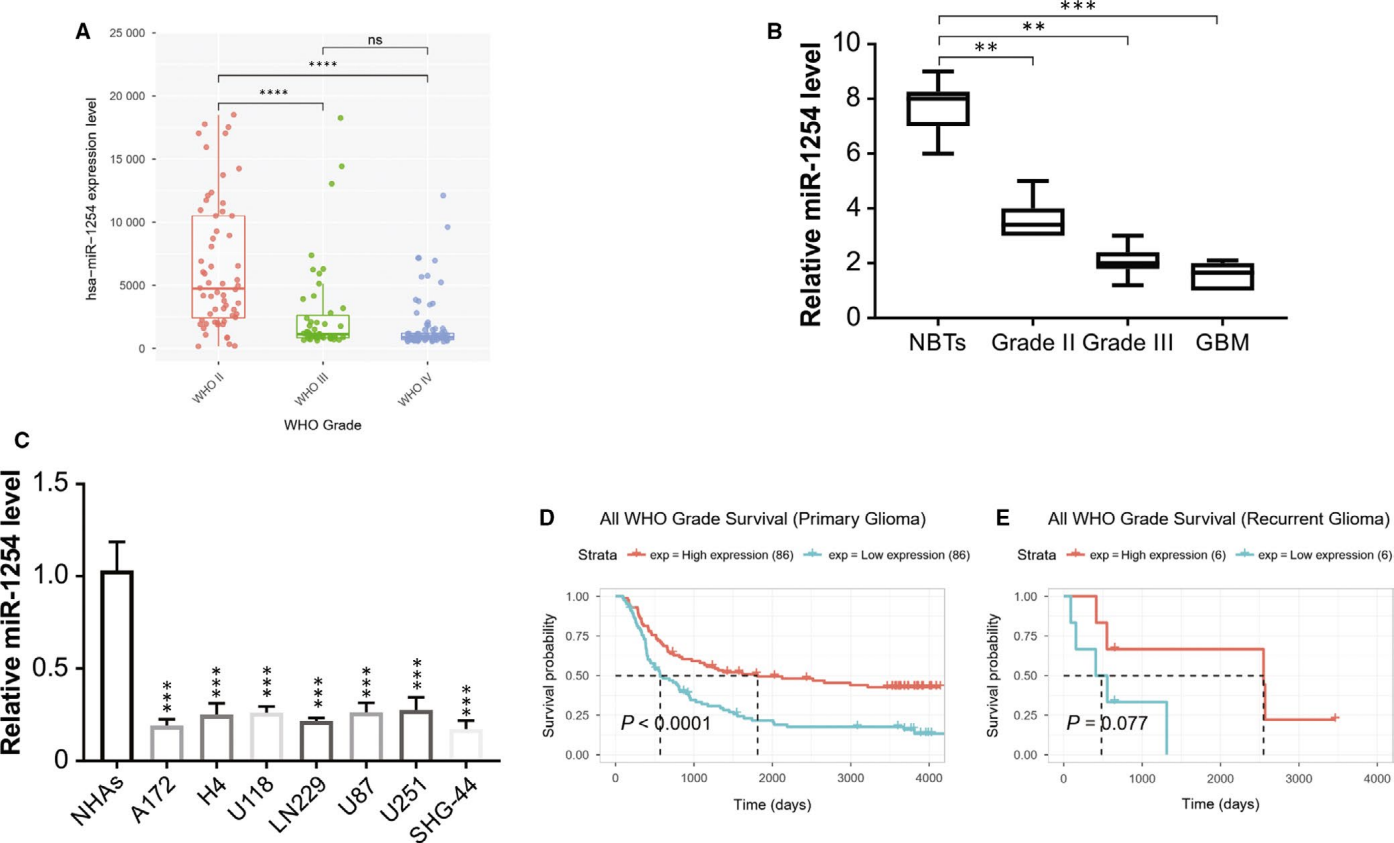
miR‐1254 expression is down‐regulated in gliomas and is negatively correlated with tumour grade. (A) CGGA database showing reduced miR‐1254 expression in high‐grade glioma tissues compared with that in low‐grade glioma tissues. (***P < .001). (B) Relative miR‐1254 expression in 30 glioma samples divided according to pathological classification (WHO II (n = 10), WHO III (n = 10) and GBM/WHO IV (n = 10)) and NBTs (n = 5). The Student's *t* test was used to analyse significant differences between groups. (C) The expression of miR‐1254 in normal human astrocytes (NHAs) and glioma cell lines was analysed by real‐time PCR. (D) and (E) Kaplan‑Meier survival analysis from CGGA database showing higher expression of miR‐1254 in patients was associated with higher survival rate in primary and recurrent glioma. Results were expressed as means ± SD of three independent experiments. **P < .01; ***P < .001

Firstly, we analysed the patterns of miRNAs expression in the CGGA database to evaluate the function of miRNAs in the initiation and progress of glioma. We found a significant decrease in miR‐1254 levels in high‐grade glioma in comparison with the low‐grade glioma (Figure 1A). Next, the level of miR‐1254 in five normal brain tissues (NBTs) and 30 gliomas were measured through real‐time PCR. Thirty glioma tissue samples were classified as per WHO as Grade II, Grade III (n = 10 each) and GBM as Grade IV (n = 10) (Figure 1B). The level of miR‐1254 was reduced significantly in glioma samples in comparison with the NBTs, particularly in GBM. To further confirm this result, RNA was extracted from the glioma cell lines A172, H4, LN229, U118 and U251, and from NHAs. The levels of miR‐1254 declined significantly in the glioma cell lines compared with that in NHAs, particularly in A172 and SHG‐44 cells (Figure 1C). Moreover, Kaplan‑Meier survival analysis form CGGA database revealed that higher expression of miR‐1254 in patients was associated with higher survival rate in primary and recurrent glioma (Figure 1D and 1). Hence, miR‐1254 expression was low in glioma and that of miR‐1254 correlated negatively with the grade of glioma.

### 3.2 Overexpression of miR‐1254 in glioma cell lines stalls their multiplication and metastasis

**Figure 2:**
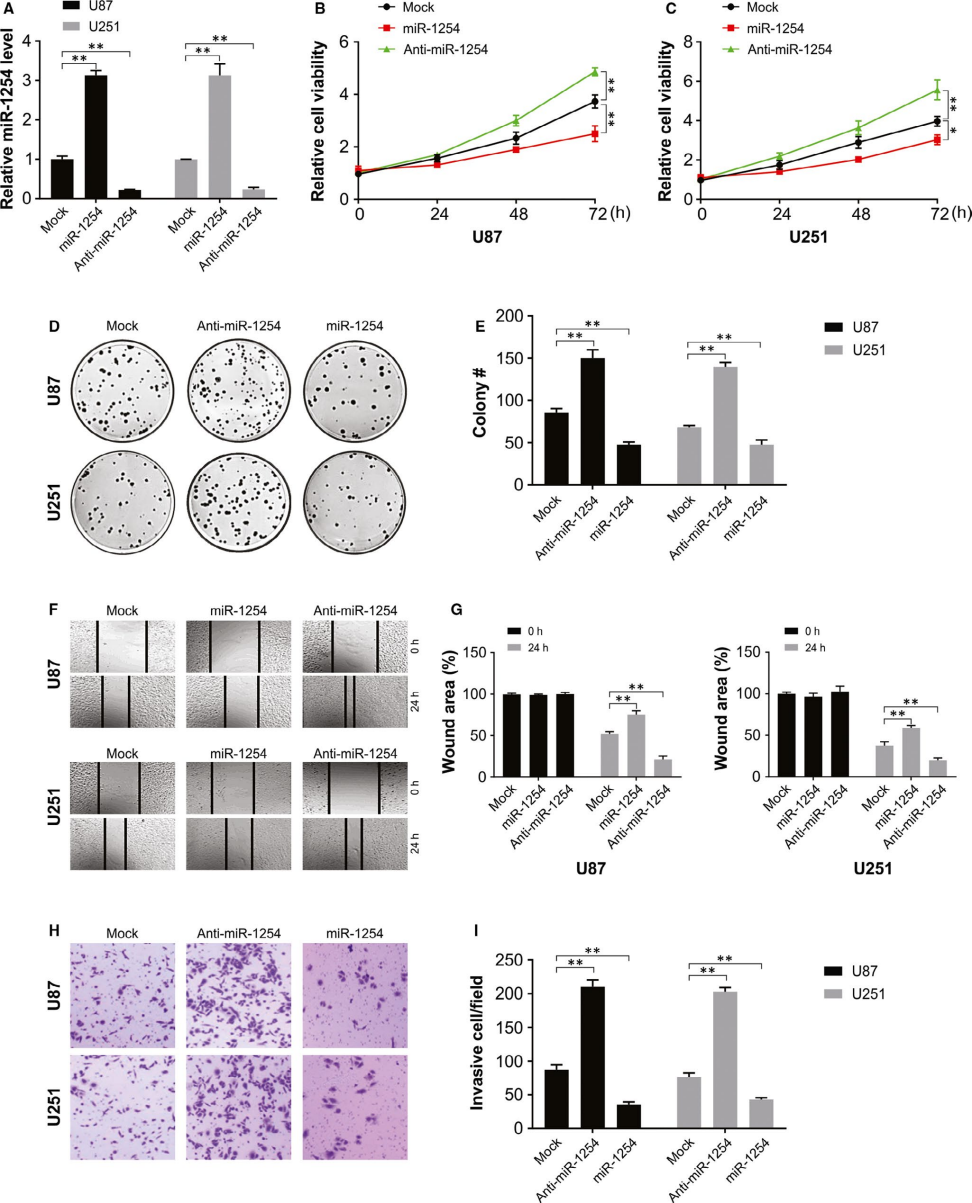
miR‐1254 inhibited the proliferation, colony formation, migration and invasiveness of glioma cells. (A) miR‐1254 overexpression and knockdown cells were successfully established. (B) and (C) Cell viability was significantly stronger in anti‐miR‐1254 group while was weaker in miR‐1254 overexpression group. (D) and (E) Colony formation number was higher in anti‐miR1254 group while was lower in miR‐1254 overexpression group. (F) and (G) Cell migration rate was remarkably higher in anti‐miR1254 group while was lower in miR‐1254 overexpression group. (H) and (I) More invaded cells were seen in anti‐miR‐1254 group while there were less invaded cells in miR‐1254 overexpression group. Results were expressed as means ± SD of three independent experiments. *P < .05; **P < .01

The transfection assay was carried out to overexpress or knockdown miR‐1254 in glioma cell lines (U87 or U251) (Figure 2A). The outcome of CCK‐8 assay showed a robust viability of cells in the anti‐miR‐1254 group, and much weaker in the miR‐1254 overexpressed group than that in the mock group (Figure 2B and 2), implicated that miR‐1254 overexpression hindered U87 and U251 cells proliferation. The colony formation assays showed miR‐1254 upregulation over 14 days led to a significant decline in the number of glioma cells than that observed for the miR‐con group (Figure 2D and 2). In both cell lines, the outcome of wound‐healing assay revealed a noticeably reduced rate of migration in miR‐1254 overexpressed group and a remarkably enhanced rate of migration in the anti‐miR‐1254 group than that in the mock group (Figure 2F and G). The transwell assay results showed that more cells in the anti‐miR‐1254 group penetrated across the membrane than in the miR‐1254 overexpression group when contrasted with the mock group (Figure 2H and I). Thus, miR‐1254 inhibited the invasive, migratory and proliferative ability of glioma cells.

### 3.3 Apoptosis induction and cell cycle arrest of U87 and U251 cells by miR‐1254

**Figure 3:**
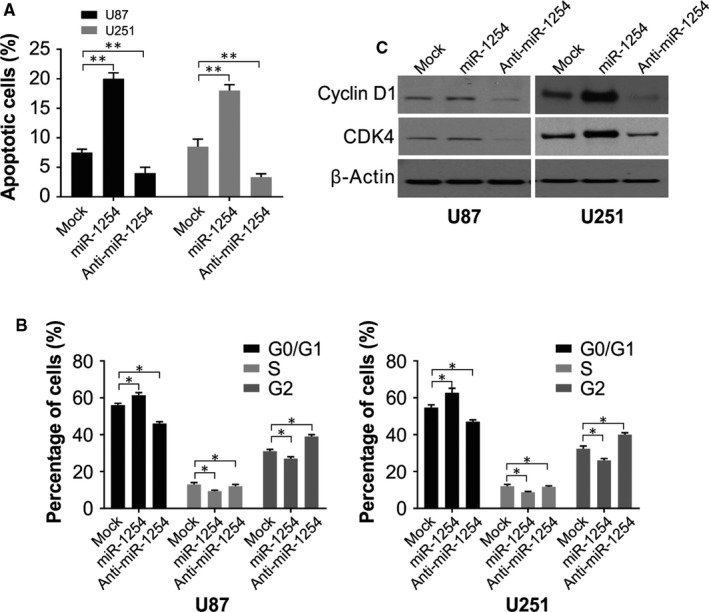
miR‐1254 promoted the apoptosis and blocked the cell cycle of glioma cells U87 and U251. (A) Results of flow cytometry showed that cell apoptosis rate was notably higher in miR‐1254 overexpression group while lower in anti‐miR‐1254 group. (B) Cell cycle progression of cells in miR‐1254 group was arrested in G0/G1 phase. (C) Western blotting of cyclin D1 and CDK4 in U87 and U251 cells after transfection with the indicated constructs. β‐Actin was used as the loading control. Results were expressed as means ± SD of 3 independent experiments. *P < .05; **P < .01

The rate of apoptosis was remarkably more in the miR‐1254 overexpressing group and reduced in the anti‐miR‐1254 group when contrasted with the mock group, indicating that apoptosis was induced in glioma cells U87 and U251 as a result of miR‐1254 overexpression (Figure 3A). Furthermore, the cell cycle distribution outcome shows an arrest of the progression of the cell cycle in G0/G1 phase in the miR‐1254 group (Figure 3B). The results of Western blotting show increased in expression of CDK4 and cyclin E in cells transfected with miR‐1254‐transfected compared with that in the controls (Figure 3C). Thus, miR‐1254 stimulated apoptosis in glioma cells and stalled the cell cycle in the G0/G1 phase.

### 3.4 Targeting relationship between miR‐1254 and CSF‐1

**Figure 4:**
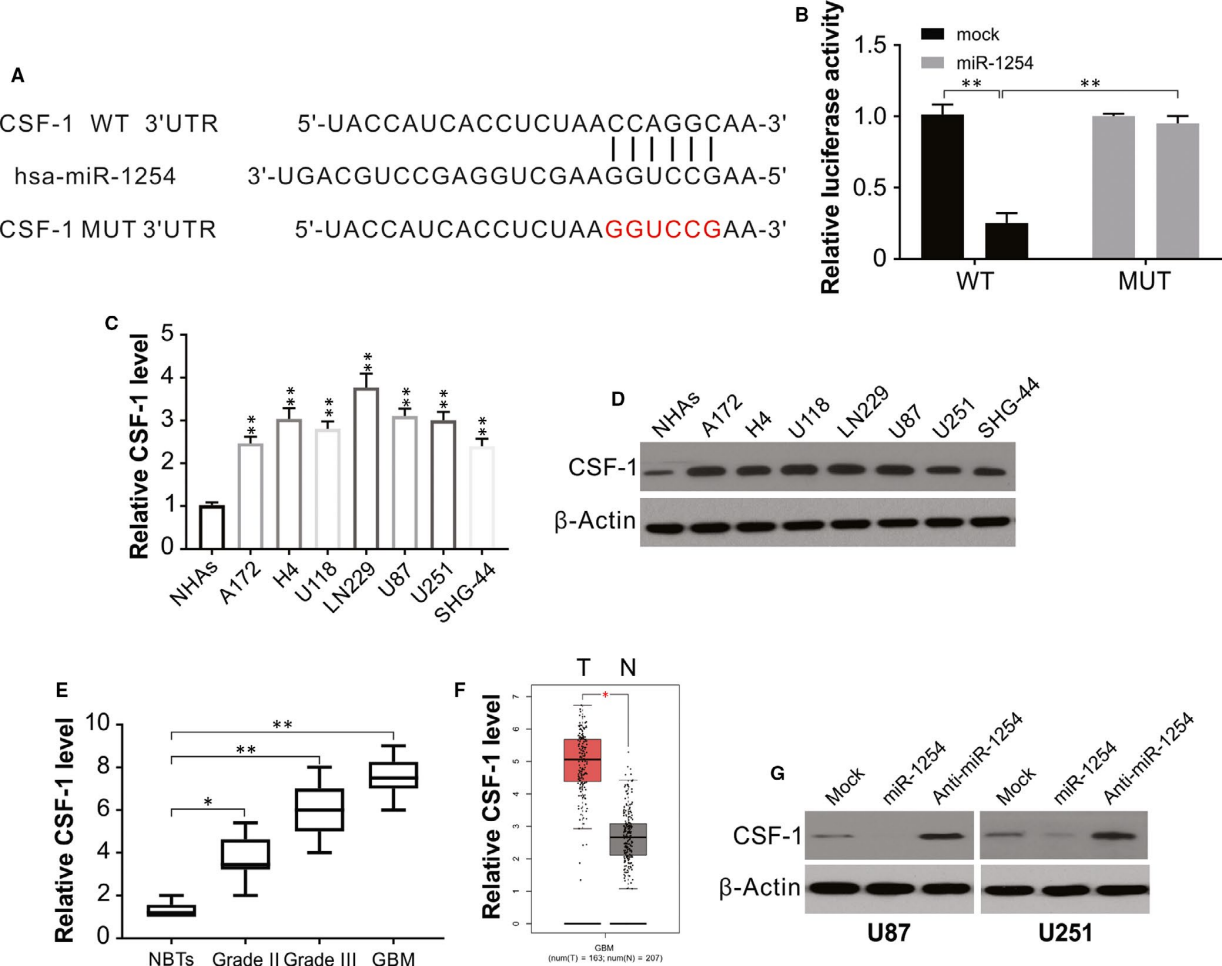
Targeting relationship between miR‐1254 and CSF‐1 in glioma. (A) The predicted base pairing in miR‐1254 and CSF‐1 from TargetScan. (B) Validation of targeting relation between miR‐1254 and CSF‐1 through dual‐luciferase reporter assay. (C) The expression of CSF‐1 was higher in glioma cell lines than that in normal cell line NHAs. (D) CSF‐1 protein expression was higher in glioma cell lines than that in normal cell line NHAs. (E) Relative CSF‐1 expression in glioma samples divided according to pathological classification and NBTs. The Student's *t* test was used to analyse significant differences between groups. (F) TCGA database showing increased CSF‐1 expression in glioma compared with normal samples. (G) Western blotting of CSF‐1 in U87 and U251 cells transfected with indicated miR‐1254 or anti‐miR‐1254. Results were expressed as means ± SD of three independent experiments. *P < .05; **P < .01

The TargetScan Database (http://www.targetscan.org/) was used to further assess the mode of action of miR‐1254's role and to predict the possible targets. The outcome of literature search and assessment of expression were considered together, CSF‐1 was chosen, and the subcloning of WT and mutated 3'‐UTR was performed in the pmirGLO vector (Figure 4A). The validation was performed through dual‐luciferase reporter assay for the targeting association between CSF‐1 and miR‐1254 (Figure 4B). As per real‐time PCR results the expression of CSF‐1 in glioma cells A172, H4, U87, U118 and U251 and was remarkably higher compared with that in normal NHAs cells (Figure 4C). This was further confirmed by western blotting that the seven glioma cell lines exhibited much high levels of CSF‐1 than that in normal cell NHAs (Figure 4D). To assess the correlation between miR‐1254 and CSF‐1 expressions in gliomas, we carried out Western blotting to determine CSF‐1 levels in NBTs, grade II and grade III, as well as GBM glioma samples. The CSF‐1 expression in tumour tissues was enhanced significantly compared with that in NBTs and correlated positively with tumour grade (Figure 4E). Next, Base on the TCGA data, we found high expression of CSF‐1 in glioma. (Figure 4F). In contrast with the mock group, the levels of CSF‐1 were dramatically lowered in the cells overexpressing miR‐1254 whereas the miR‐1254 knockdown cells exhibited enhanced protein levels (Figure 4G), indicating that the expression of CSF‐1 was regulated by miR‐1254 in U87 and U251.

### 3.5 CSF‐1 medicates the function of miR‐1254 in glioma cells

**Figure 5:**
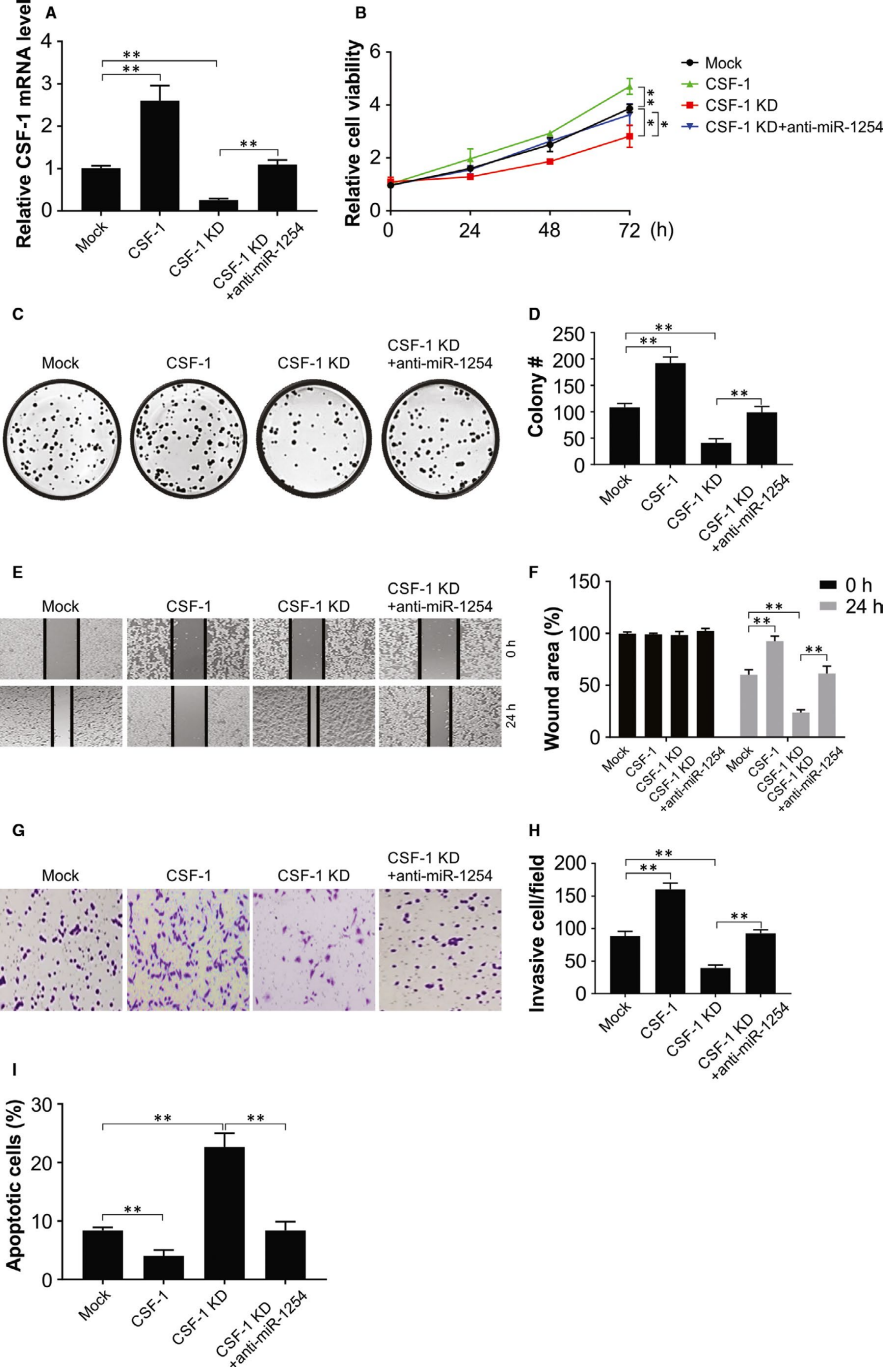
CSF‐1 promoted the proliferation, colony formation, migration and invasion of glioma. (A) U87 cells of CSF‐1 overexpression and knockdown were analysed by real‐time PCR. (B) CCK‐8 assay showed that cell viability was significantly stronger in CSF‐1 overexpression group while was weaker in CSF‐1 knockdown group, and there was no significant difference between CSF‐1‐KD + anti‐miR‐1254 group and mock group. (C) and (D) Colony formation assay showed that colony number in CSF‐1 overexpression group was remarkably higher, whereas that in CSF‐1 knockdown group was much lower, and there was no significant difference between CSF‐1‐KD + anti‐miR‐1254 group and mock group. (E) and (F) Wound‐healing assay showed that cell migration rate in CSF‐1 overexpression group was remarkably higher, whereas that in CSF‐1 knockdown group was much lower, and there was no significant difference between CSF‐1‐KD + anti‐miR‐1254 group and mock group. (G) and (H) More invaded cells were observed in CSF‐1 overexpression group while the number of invaded cells in CSF‐1 knockdown group was significantly lower compared with the mock group, and there was no significant difference between CSF‐1‐KD + anti‐miR‐1254 group and mock group. (I) Cell apoptosis rate was notably higher in CSF‐1 knockdown group while lower in CSF‐1 overexpression group, and there was no significant difference between CSF‐1‐KD + anti‐miR‐1254 group and mock group. Results were expressed as means ± SD of three independent experiments. *P < .05; **P < .01

The real‐time PCR results showed that although the level of CSF‐1 mRNA was promoted by CSF‐1 cDNA, it was stalled by CSF‐1 shRNA (Figure 5A). Besides, the expression of CSF‐1 was rescued by cotransfection of anti‐miR‐1254. The results of CCK‐8 show that the glioma cell viability was more robust in the CSF‐1 overexpression group but was significantly reduced in the group with CSF‐1 knockdown compared to that in the mock group (Figure 5B). Furthermore, the stalling effect of CSF‐1 shRNA on cell viability was impeded due to miR‐1254 knockdown. Furthermore, colony formation and CCK‐8 assays showed that the CSF‐1 significantly annulled the effects of miR‐1254 on cell proliferation (Figure 5C and 5). The wound‐healing assay shows that the rate of cell migration in CSF‐1 overexpressing group was significantly enhanced, while that in the group with CSF‐1 knockdown declined noticeably compared to the mock group (Figure 5E and 5). Concurrently, no significant difference between CSF‐1 shRNA + anti‐miR‐1254 group and the mock group was observed. In transwell assay, the CSF‐1 overexpressing group exhibited more invaded cells whereas a smaller number of cells moved across the membrane in CSF‐1 knockdown group in comparison with the mock group (Figure 5G and H), and anti‐miR‐1254 impeded this hindering effect of CSF‐1 shRNA on cell invasion. Therefore, CSF‐1 promoted the migration and invasion of U87 cells, and the negative effect of CSF‐1 shRNA on cellular metastasis could be reversed by anti‐miR‐1254. Therefore, a negative correlation exists between CSF‐1 and miR‐1254 with respect to their effects on functions of glioma cells.

To better assess the effect of CSF‐1 on cell functions, the CSF‐1 overexpressing and the knockdown cells were studied for apoptosis and cell cycle distribution. Indeed, CSF‐1 overexpression reduced the rate of apoptosis, while of CSF‐1 inhibition enhanced the apoptosis of glioma cells, which was disrupted by anti‐miR‐1254 (Figure 5I). Hence, CSF‐1–induced tumour development was an activity exactly contrary to the role of its targeting regulator, miR‐1254.

### 3.6 The in vivo development of glioma cell U87 is suppressed by miR‐1254

**Figure 6:**
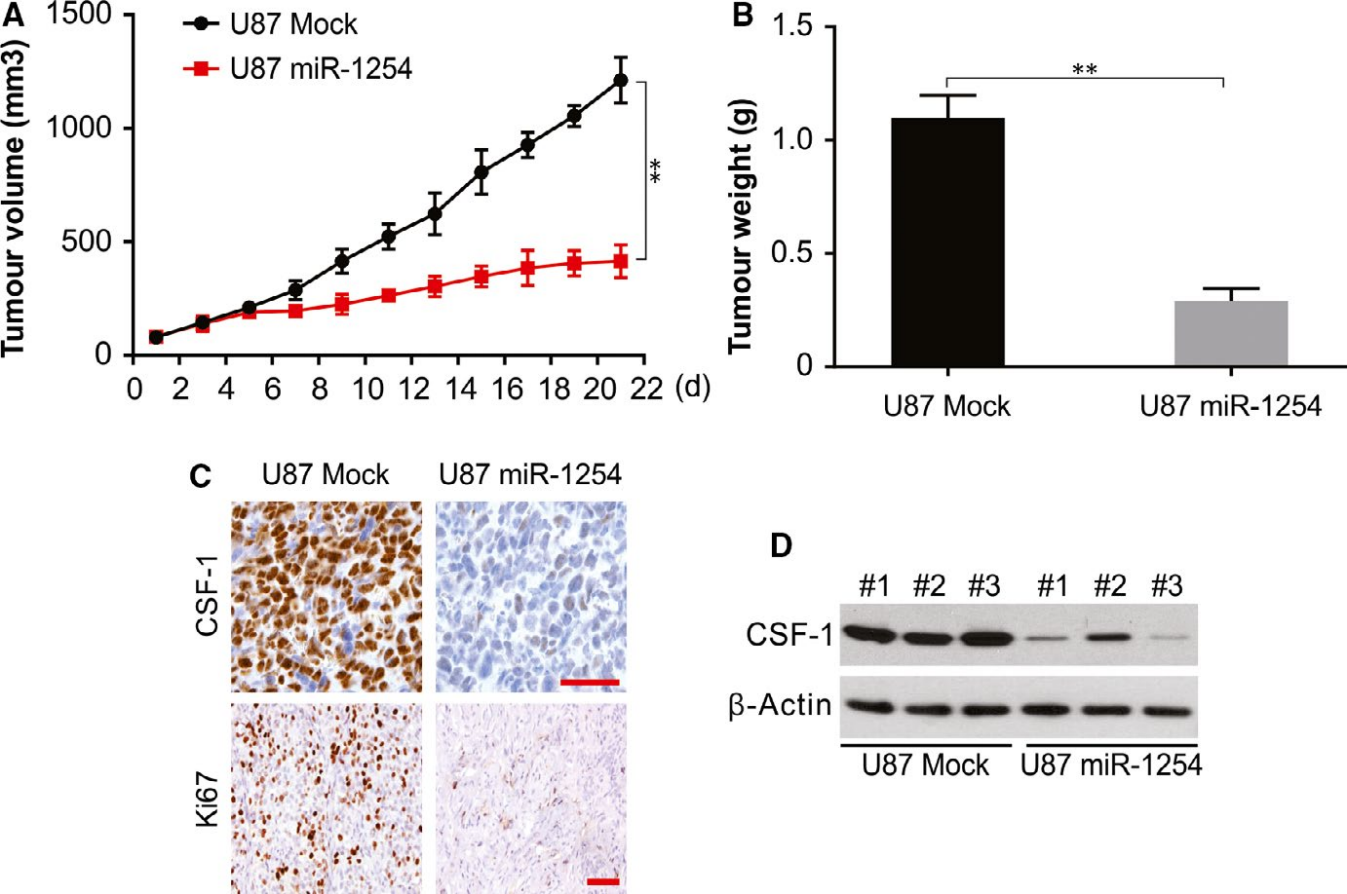
miR‐1254 suppressed tumour formation of glioma cell U87 in nude mice. (A) and (B) Tumours in nude mice were remarkably smaller in miR‐1254 overexpression group compared with the mock group. (C) Representative immunohistochemical images of CSF‐1 and ki‐67. Scale bar: 25 μm. (D) Western blotting of CSF‐1 in indicated group. Results were expressed as means ± SD of three independent experiments. **P < .01

Post‐modulation of miR‐1254 expression in U87 cells with AgomiR‐1254, nude mice were subcutaneously injected with these cells, and untreated U87 cells were injected in mice as mock group and tumour development was observed as mentioned in the methods section. An obvious attenuation of tumour development in U87‐miR‐1254 group was seen than in the mock group (Figure 6A‐B). The immunohistochemical assay revealed significantly down‐regulated levels of CSF‐1 and Ki67 in the tumour tissues of nude mice treated with miR‐1254 compared to that in controls (Figure 6C). Furthermore, tumours from miR‐1254 cells remarkably inhibited CSF‐1 expression, which was also confirmed by western blotting (Figure 6D). Thus, the results indicate a noticeable inhibitory effect of miR‐1254 on CSF‐1 and therefore may possibly act as therapeutic options for glioma patients.

## 4 DISCUSSION

As posttranscriptional gene regulators, miRNAs participate in several processes of cancer, such as angiogenesis, proliferation and invasion.^34^, ^35^ Human cancers, including gliomas, characteristically exhibit abnormal expression of miRNAs, in contrast to the healthy tissues.^36^, ^37^ MiRNAs act as suppressors of oncogenes or tumours by regulating the expression of the target genes.^38^ They can also indicate survival and prognosis in several cancers by acting as biomarkers.^39^ Thus, for a clearly diagnosis and accurate treatment of patients with malignancies, the identification of targets of specific miRNAs involved in tumorigenesis is imperative. For instance, the function of miR‐1254 on gliomas has not yet been fully examined.

In this study, miR‐1254 was found to be down‐regulated and had an inverse relation with the grade of glioma as per CGGA database analysis. We further confirmed a dramatic decrease in expression of miR‐1254 both in glioma cells and in tissue samples. MiR‐1254 overexpression stalled the progression of glioma, induced apoptosis in the tumour cell and blocked the cell cycle in vitro, as well as repressed in vivo tumour growth. We also confirmed CSF‐1 as the direct target of miR‐1254 through dual‐luciferase reporter assay, and that overexpression of CSF‐1 invigorated viability and metastasis of glioma cells. Further, the glioma therapy could be highly benefitted by improving the suppression effect of miR‐1254 on glioma cell progression. The potential of miR‐1254 to act as a biomarker should be further examined for the early diagnosis of gliomas.

Cell behaviour, such as proliferation and invasion are associated with the expression of miRNAs.^40^ For instance, glioma cell malignancy was seen to be inhibited by miR‐105 and miR‐592, whereas glioma cell metastasis is promoted by others like miR‐21 and miR‐183.^41^, ^42^, ^43^, ^44^ The suppressive action of miR‐1254 in malignancies including gastric cancer, lung cancer and laryngeal cancer has been reported by several studies.^45^, ^46^ With this as a basis, we attempted to determine the role of miR‐1254 in human glioma. We observed a repression of miR‐1254 in human glioma tissues and cells and a robust suppressive effect of overexpressed miR‐1254 on progression and tumour growth in glioma cells. Hence, we implicate a tumour‐inhibitory activity of miR‐1254 and its involvement in glioma proliferation.

We next using bioinformatics analysis to explore the target genes of miR‐1254, and identified CSF‐1 as a potential target. Overexpression of CSF‐1 has been reported in cancers of ovary, breast and liver, and is inversely related to prognosis and survival rate in these patients. We could discern miR‐1254 as a primary mediator of proliferation in glioma that targets CSF‐1 directly. We found significantly enhanced CSF‐1 expression in glioma specimens, and also showed both in vitro and in vivo, that increased expression of miR‐1254 repressed CSF‐1 level, indicating an inverse relationship between them. We also showed a specific binding of miR‐1254 to CSF‐1 3′‐UTR. Considering these in conjunction, as per these findings miR‐1254 has a vital role in inhibiting proliferation of glioma cells via the CSF‐1 suppression. We also confirmed the target association between miR‐1254 and established CSF‐1 knockdown as well as overexpressing cells to determine its impact on cellular processes in glioma. The outcome revealed that CSF‐1 overexpression promoted glioma cell propagation. Therefore, a negative relationship exists between CSF‐1 and miR‐1254 and their contrasting effects on initiation and advancement of glioma. Nevertheless, our study still had few insufficiencies.

There are some limitations in the current study. First, only xenograft mouse model was used in this study. Orthotopic cancer mouse model will be used to test the effect of miR‐1254 on glioma. Second, more clinical samples should be used to confirm the function of miR‐1254. Third, the following study will focus on the combination effect of miR‐1254 with TMZ or other clinical drugs to treat glioma.

In summary, this is the first evidence for the association between miR‐1254 and human glioma progression, with a negative correlation between miR‐1254 expression and the histological grade. We further revealed the tumour suppressing activity of miR‐1254 on the growth of human gliomas. Our newly confirmed miR‐1254/CSF‐1 axis aids in delving into the mechanisms of glioma development and may help formulating more robust molecular targeting therapy for human gliomas.

## CONFLICT OF INTEREST

The authors declare that they have no conflict of interest.

## AUTHOR'S CONTRIBUTION

XL and YC performed the experiments. XL, SK and YC designed the research study. XL analysed the data. XL wrote the manuscript. All authors reviewed and approved the manuscript.

## Data Availability

The data used to support the findings of this study are available from the corresponding author upon request.
